# Acetylcholinesterase Biosensors for Electrochemical Detection of Organophosphorus Compounds: A Review

**DOI:** 10.1155/2013/731501

**Published:** 2013-12-09

**Authors:** Vikas Dhull, Anjum Gahlaut, Neeraj Dilbaghi, Vikas Hooda

**Affiliations:** ^1^Department of Bio & Nanotechnology, Guru Jambeshwar University of Science & Technology, Hisar 125001, India; ^2^Centre for Biotechnology, Maharshi Dayanand University, Rohtak 124001, India

## Abstract

The exponentially growing population, with limited resources, has exerted an intense pressure on the agriculture sector. In order to achieve high productivity the use of pesticide has increased up to many folds. These pesticides contain organophosphorus (OP) toxic compounds which interfere with the proper functioning of enzyme acetylcholinesterase (AChE) and finally affect the central nervous system (CNS). So, there is a need for routine, continuous, on spot detection of OP compounds which are the main limitations associated with conventional analytical methods. AChE based enzymatic biosensors have been reported by researchers as the most promising tool for analysis of pesticide level to control toxicity and for environment conservation. The present review summarises AChE based biosensors by discussing their characteristic features in terms of fabrication, detection limit, linearity range, time of incubation, and storage stability. Use of nanoparticles in recently reported fabrication strategies has improved the efficiency of biosensors to a great extent making them more reliable and robust.

## 1. Introduction

At present pesticides play a major role in agriculture. Pesticides have the insecticidal property due to which they are in great use [1, 2]. But human health and the surroundings are affected by these pesticides as they contain the toxic compounds. These toxic compounds are hazardous as they can accumulate in grains, vegetables, fruits, and so forth, percolate in soil, and finally lead to water contamination [3, 4]. The concentration of these toxic compounds in the environment is increasing day by day with an exponential rate. Organophosphorus (OP) constitutes one of the important classes of toxic compounds which can cause headache, drowsiness, confusion, depression, irritability, disorientation, impaired memory and concentration, speech difficulties, eye pain, abdominal pain, convulsions, respiratory failure, and serious neurological disorders [5–10]. The EPA lists organophosphates as very highly toxic to bees, wildlife, and humans [1]. These OP pesticides inhibit the enzyme acetylcholinesterase (AChE, EC 3.1.1.7) which is involved in the proper functioning of the central nervous system (CNS) of the humans. Due to this inhibition of the enzyme AChE, acetylcholine (ACh) neurotransmitter accumulates in the body which interferes with the muscular responses and finally leads to respiratory problems, myocardial malfunctioning, and even death [11, 12]. The toxicity of different pesticides depends upon the chemical structure of the pesticides [12, 13]. The repeated low level exposure to OP compounds leads to the acute effect on the health of humans. The contamination of soil and food due to these pesticides has caused a serious concern, so it is necessary to monitor their increasing concentration in the food products of daily use. Soil is known to be a natural purifier in which the OP pesticides along with water interact with the soil particles and do not contaminate ground water, but by the time some of the OP pesticides come forward such as organochlorine pesticides which can even percolate even through the soil and contaminate both ground and surface water. Many rules and regulations have been made on the international level to reduce the contamination of ground and surface water. Regulatory limits and the guideline levels are also there for permissible residues in drinking water [14].

It is necessary to develop the methods which are fast, sensitive, and reliable for the detection of OP pesticides in fruits, vegetables, water, and so forth [15]. Conventional analytical methods to monitor the concentration of these acute toxic compounds include capillary electrophoresis [16], colorimetry [17], gas chromatography (GC) [18], mass spectrometry (MS) [19], thin layer chromatography [20, 21], and high performance liquid chromatography (HPLC) [22]. The above said methods have some limitations, that is, sample preparation which is hectic and time consuming; requiring expensive equipments and trained manpower; less economical; and so forth. To overcome the above problems, development of biosensor is being encouraged. They are simple, sensitive, of low developmental cost, and user friendly; a normal person can handle it easily.

The present review describes and discusses the use of AChE biosensors for detection of OP compounds and measurement of toxicity level in different samples.

## 2. AChE Based Catalysis

AChE belongs to the family of carboxylesterase (EC number 3.1.1.7.). It is serine protease and stabilises level of acetylcholine (neurotransmitter) by catalysing the conversion of acetylcholine to thiocholine. AChE is concentrated at neuromuscular junctions and cholinergic brain synapses. When the enzyme is present in the active form it terminates synaptic transmission. AChE is highly efficient and catalyses the breakdown of ACh in microseconds keeping the synaptic cleft clear as to avoid the collision of the messages. AChE has two active subsites, anionic and esteratic subsite. Acetylcholine mediates messages between the nerves which is responsible for muscle contraction. When ACh is released from the nerve into the synaptic cleft, it got recognised by ACh receptors present on the postsynaptic membrane which further transmits signal. Along with the ACh receptors AChE is also present on the postsynaptic membrane which helps in the termination of the signal transmission by hydrolysing ACh. On hydrolysis, ACh split into two products one is choline and the other is acetic-acid. Choline and acetic-acid are recycled by the body to again form acetylcholine to maintain the reserves of neurotransmitters so that they can be used by the body again during the time of need. In the presence of inhibitor (OP compound), which forms covalent bond with serine present on the active site of AChE, leads to inactivation of the enzyme [54, 55], and the muscles involved do not relax, leading to paralytic conditions. The intensity of inhibition of AChE is proportional to the concentration of OP compound, that is, inhibitor, and is also exploited as principle of detection method for concentration of OP compounds [55–58].

## 3. Basic Principle of Biosensors

**Figure 1:**
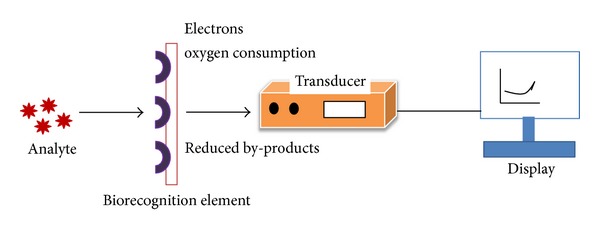
Basic principle of electrochemical biosensor.

Biosensor comprises basically of three elements, that is, biological recognition element, transducer, and signal detector as shown in Figure 1. The biological recognition element must be extremely specific to the analyte for the accurate detection of the analyte in different samples. As recognition element and analyte come in close proximity to each other the chemical changes take place in the form of the generation of electroactive species, reduced forms of by-products, consumption of oxygen, and so forth [59]. These changes are detected and displayed on controlling system.

### 3.1. Principle of OP Biosensor Based on Inhibition Mechanism of AChE

**Figure 2:**
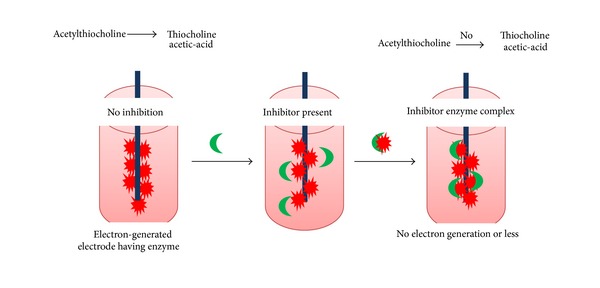
Principle of AChE inhibition-based OP biosensor.

**Scheme 1:**
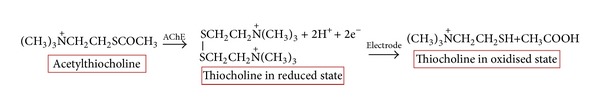
Reaction involved in generation of electrochemical response biosensor.

The sensitivity of biosensor relies on the biorecognition layer which catalyses the reaction. The product/by-product further or itself acts as signal which is directly or inversely proportional to the analyte concentration. In case of AChE inhibition based OP biosensors, the signal generated is inversely proportional to the concentration of OP compound or, in other terms, we can say that increased concentration of OP compound leads to weak signals. The AChE biosensor basically works on the inhibition effect. The biosensor in which the AChE is used as the biorecognition element can detect the toxic organophosphates along with the others such as carbamate pesticides, nerve agents, and several other natural toxins [60, 61]. Some drugs can also be detected with the help of such biosensors [62]. If the inhibitor is not present in the sample then the acetylthiocholine will be converted into the thiocholine and the acetic-acid. as shown in Scheme 1. But if the inhibitor is present in the sample then the concentration of thiocholine is decreased or no thiocholine and acetic-acid is produced, in other words it completely inhibits the conversion as shown in Figure 2 [63]. Under the influence of applied voltage thiocholine is oxidised. The anodic oxidation current is inversely proportional to the toxic compound present in the sample and the time of exposure.

In the beginning, AChE biosensors were not considered as reliable tools, but with time the advances in fabrication strategies and methods of enzyme purification and its stabilization have overcome the drawbacks related to accuracy, sensitivity, and reliability [64].

## 4. Fabrication of AChE Based OP Biosensor

**Figure 3:**
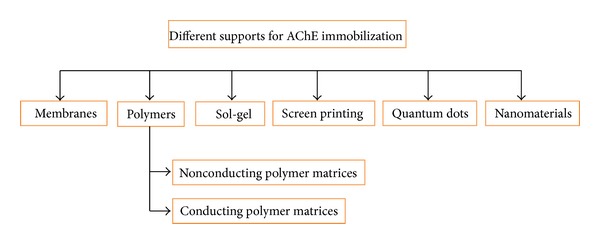
Different supports available for fabrication of working electrode.

In AChE biosensor the working electrode is prepared by attachment of enzyme on different supports. The supports may be matrices, screen-printed electrodes, semiconductors such as Quantum dots (QD), nanomaterial, and so forth [127], as shown in Figure 3. After immobilization of enzyme onto a particular support, conformational changes take place which finally affect the sensitivity, stability, response time, and reproducibility. A variety of methods are available for immobilization of enzymes including physical adsorption, physical entrapment, covalent coupling, self-assembly monolayer, oriented immobilization, and electropolymerisation. Physical adsorption includes the formation of weak bonds such as the Van der Waals forces, and the electrostatic interactions take place between the enzyme and the support that has an advantage of retaining the activity of immobilized enzyme and method is economical. The drawback associated with this method is the leakage of enzyme [79]. In physical entrapment, AChE enzyme is confined within the gel, the matrices, or in the membranes and used for fabrication of working electrode. This is a one-step procedure which is carried out at low temperature, is simple and cheap, without hampering the activity to enzyme. This method also suffers from leaching of enzyme, nonspecific immobilization, and lower reproducibility. In covalent coupling, stable covalent bond is formed between the support and the enzyme that prevents leaching of enzyme, enzyme is in direct availability for interaction with the analyte that further leads to quick response time. But this method involves a high amount of enzyme usage, is prone to denaturation, is also expensive, and involves complex procedures [42, 96]. In case of self-assembled monolayer (SAM) the molecules are organised in the form of monolayer. These molecules have the head group and also a tail group having functional groups; head group has affinity towards the substrate. This layer is easy to prepare, molecules are present in the ordered manner, and size is also within the range of nanoscale. Drawbacks of this method are includes difficulties in reproduction and fouling of electrode takes place with time due to the weakening of interaction between the enzyme and the electrode [115, 116]. Oriented immobilization is among one of the new methods which can be used. In this method the particular functional groups of the enzymes are exploited and it is possible to orient the active site of the enzyme towards the analyte. This technique requires less quantity of enzyme with specific control over the orientation [117]. Electropolymerization is also one of the possible methods for the immobilization of the AChE enzyme in which the electric field is used for the polymerization.

### 4.1. Membranes Used in Fabrication of OP Biosensor

In membrane based AChE biosensors the enzyme is immobilized on the suitable matrices. The membranes which are used as support for immobilization can be natural or artificial. The enzyme is confined to the semipermeable membrane which will allow the passage of the substrate through it. The sensitivity and the selectivity of the membrane based biosensors can be enhanced due to the biocompatibility of the artificial membranes. Different supports have been used for the immobilization of enzyme (Table 1), such as nylon and cellulose nitrate [23], glass/sol-gel/polyvinylidene fluoride [24], hybrid mesoporous silica [25], poly-(acrylonitrile-methylmethacrylate-sodium vinylsulfonate) (PAN) [26, 27], cellophane [28], poly(2-hydroxyethyl methacrylate) membrane [29], polyvinyl alcohol(PVA)/SbQ [30], polyacrylamide [31], bio-immunodyne membrane [118], Si_3_N_4_/Ti layer [32], pore glass/H^+^ membrane electrode [33], and hybond N^+^ membrane [34]. The artificial membranes are selective for the different biomolecules, and as they are highly flexible the response can be enhanced. Membranes are durable and stable on a wide range of pH. The above biosensors suffer from the problem of membrane fouling. The pores of semipermeable membranes are blocked which may lead to hindrance in the passage of solute.

### 4.2. Polymers Used as Immobilization Support

Polymers can also be used as the support for the enzyme immobilization. The physical and chemical properties of the polymers vary in the wide range which can be exploited for the sensor development [119]. Scince polymer supports are flexible, biologically compatible and of low cost, they have advantage over the other supports. They can be used as free standing film for the biosensor fabrication [120].

#### 4.2.1. Nonconducting Polymer Matrices for Enzyme Immobilization

The nonconducting polymer supports can easily be prepared in the lab. The variety of the functional groups can be generated on these supports by the chemical treatment. The functional groups of interest according to the particular enzyme can be synthesized on such supports. The life of the enzyme can also be enhanced by this method as it provides a microenvironment to the enzyme and can be stored for a long duration. But there are drawbacks with nonconducting polymers, being a support it acts as a barrier between electron and transducer which thus influences the sensitivity of electrode due to which working of electrode is affected. Some supports which are used for immobilization of enzymes are (Table 2) multiwalled carbon nanotubes (MWCNTs)/PAN/Pt electrode [26], PAN/gold nanoparticles (AuNPs) decorated Pt electrode [27], mesocellular silica foam (MSF)-PVA/glassy carbon electrode (GCE) [35], PVA-SbQ polymer decorated screen-printed electrode (SPE) [36], PVA-SbQ/Pt electrode [30], polyamidoamine (PAMAM)- Au/carbon nanotubes (CNTs)/GCE [37], MSF/PVA/GCE [38], nylon net [39], PVA/ azide-unit water pendant (AWP) [121], and CoPC modified PVA-AWP electrode [40].

#### 4.2.2. Conducting Polymer Matrices Used for Enzyme Immobilization

The conducting polymers are the polymers which are synthesized by the chemical and the electrochemical method. The properties of these polymers can easily be adjusted according to the need such as the thickness of film, functionalization, conductivity, and so forth. They can also be used for the enzyme entrapment during electropolymerization and used in the uniform covering of the electrode surface having substrate of any shape and size with the help of the polymer film [122, 123]. Different supports which are used for immobilization of enzymes are (Table 3) poly-(acrylamide)/pH electrode [31], polyethylenimine (PEI)/GCE [41], PEI/SPE [42], mercaptobenzothiazole/polyaniline (PANI)/Au electrode [43], PANI/CNTs coated with single stranded DNA (ssDNA)/Au electrode [44], AuNP-polypyrrole (PPy) nanowire/GCE [45], PPy and PANI copolymer dopped MWCNTs/GCE [46], Silk fibronin matrix [47], CS/ALB/GCE [48], PB/GCE [49], GnPs/Chitosan/GCE [50], polymeric enzyme electrode [51], ZrO_2_/SPE [52], and Gold (Au) nanoparticles/poly(dimethyldiammonium chloride) (PDDA) protected Prussian blue (PB) matrix [53]. The conducting polymers suffer from demerits of high cost, difficult in processing, lack of mechanical stability after doping, difficult to fabricate, short life span, and so forth.

### 4.3. Sol-Gel Base AChE Immobilization

Sol-gel is one of the important supports which can be used for the enzyme immobilization. The first and foremost important property of the sol-gel support is that the pore size can be adjusted according to the need. They are also chemically inert, do not show swelling in the aqueous medium, and have photochemical and thermal stability. The antibodies and the enzymes can especially be immobilized and do not allow the leakage of the enzyme in the medium. Some of the accountable demerits include denaturation of biomolecules taking place at high acidic condition and/or high alcohol concentration. The protocols used for the sol-gel film formation are not amenable for coating the curved surfaces of substrates such as optical fibers; sufficient signals require a high level of biomolecules in sol-gel thin films but it is not possible in the case of proteins that are insoluble or aggregate in the alkoxy silane solution. Sol-gel supports used for immobilization of enzyme (Table 4) are sol-gel/TMOS [65], sol-gel/glass [66], silica sol-gel (SiSG) [67], TMOS/sol-gel [68, 69], chromoionophore/sol-gel [70], Al_2_O_3_/sol-gel [71], sol-gel matrix/TCNQ [72], AuNPs-SiSG [73], alumina/sol-gel [74], sol-gel/bromothymol blue [75], Zn(oxide)/sol-gel [76], Si/sol-gel [77], and sol-gel/carbon electrode [78].

### 4.4. Screen Printing Technique

Screen-printing involves the immobilization of the biological molecules or the biological receptor in their active form. Due to the binding of the molecule in the active form, the analytical changes take place which will affect the sensitivity and the performance of the sensor developed. The necessary action must be taken for the enhancement of the selectivity, sensitivity, exposure time, and so forth. Supports used for immobilization of enzyme (Table 5) are TMOS/sol-gel/SPE [68], Al_2_O_3_/sol-gel/SPE [71], sol-gel/TCNQ/modified SPE [72], SPE/TCNQ/Graphite electrode [79], CoPC/SPE [80], phenylenediamine/carbon/CoPC SPE [81], graphite-epoxy/SPE [82], glutaraldehyde vapour/SPE [83], PVA-SbQ polymer/SPE [36], SWCNT-CoPC/SPE [84]. TCNQ modified graphite [85], Au electrode [86], screen printed carbon electrode [87], and PET chip SPE [88]. Screen-printing is unstable, has high cross-sensitivity towards anion, and limited life span.

### 4.5. Quantum Dot as Immobilization Support for AChE

Quantum dots are highly luminescent photostable fluorophore. QDs are the semiconductor particles that have all the dimensions confined to the nanometre scale [124]. They have been used in biosensors as they have their great size dependent properties and are dimensionally similar with the biological molecules which are used for immobilization [125, 126]. QDs can even be coupled with the variety of biological molecules due to which they are important in the sensing and development of the sensitive sensors. They suffer from demerits such as large size (10 to 30 nm) and blinking behaviour if no emission interrupts longer periods of fluorescence. The supports which are used for the immobilization of the enzymes are (Table 6) supports used for immobilization of enzyme: CdTe QDs/AuNPs/CHIT/GCE [73], CdTe QDs/Au electrode [89], poly(allylamine hydrochloride)/CdTe QDs/glass electrode [90], Mn:ZnSe d dots [91], and CdTE QDs/Au electrode [92].

### 4.6. Nanomaterial Based AChE Immobilization

To improve the reliability of electrochemical based technique, researchers have been exploring the possibilities of new materials for improving the properties of transducers. Nanoparticles are proving to be a boom in the field of biosensing due to their invaluable properties such as large surface area, high conductivity, good catalytic property, and so forth. The rate of electron transfer is enhanced to a great extent. They can be synthesized in the laboratory and even their particle size can be adjusted according to the need. The carbon nanotubes are in regular use nowadays such as Single Walled Carbon Nanotubes (SWCNTs) and Multiwalled Carbon Nanotubes (MWCNTs). These carbon nanotubes are highly conductive and have large surface area. Different supports used for immobilization of enzyme (Table 7) are AuNPs-CaCO_3_/Au electrode, Iron(Fe) NP/MWCNTs/Au electrode, FeNP/MWCNTs/indium tin oxide (ITO) electrode, AuNPs/PB/GCE [93], MWCNTs-Au nanocomposites/GCE [94], ZrO_2_/CHIT/GCE [95], Au-Pt bimetallic NPs/GCE [96], AuNPs/GCE [97], AuNPs-MWCNTs/GCE [98], PB/CHIT/GCE [99], TiO_2_ graphane/GCE [100], graphite-nanoplatelet CHIT composite/GCE [101], calcium carbonate-CHIT composite/GCE [102], CdS-decorated graphene nanocomposite [103], CHIT-GNPs/Au electrode [92], MWCNTs-CHIT/GCE [104], AuNPs/Au electrode [105], PbO_2_/TiO_2_/Ti [106], PB-CHIT/GCE [107], Er-GRO/Nafion [108], SWCNT modified FGE [109], Au-PtNPs/3-aminopropyltriethoxysilanes (APTEs)/GCE [110], CNT web modified GCE [111], PAN-AuNPs [112], CdTe AuNPs Film [113], and SiSG-AuNPs [114].

## 5. Conclusion and Future Prospects

It is clear from the comprehensive review presented above that the AChE based OP biosensor is an important research field, with lots of applications in environmental monitoring, human health concern, and food industries. With the development of the selective biorecognition elements the high throughput screening of analyte is now possible in a reliable manner in a fraction of seconds. The large number of samples can be screened with ease and accuracy. The oxidising and reducing ability of the biorecognition element has made electrochemical biosensor the most appropriate tool for the detection purpose over the other available methods [127]. The electrochemical biosensors have the unique ability to convert the catalytic signal into the quantifiable digital signal using microfabrication electronics. Nanoparticles are proving to be most eligible in fabrication of different working electrodes. The nanomaterials can be easily synthesized in the laboratory according to the need in respect to their size and dimensions. The conductivity of nanomaterials is high due to efficient electron transfer channels developed with respect to the other supports used. The self-life of the AChE biosensor can also be increased by using nanoparticles based electrodes. A vast variety of working electrodes for the sensor development can be fabricated for the improved detection of OP compounds in different samples. The on spot detection is also an important parameter for the biosensors which is possible due to the screen-printing technology. Screen-printed biosensors can be fabricated in miniaturization form for on-site rapid monitoring of the analyte. But till now the commercialization of the biosensors has not been possible due to the high cost of the enzyme in the market. Less work has been done on the validation of the enzymatic biosensors with respect to the real samples. Many interfering compounds are present in the sample and can hamper the sensitivity of the biosensor. The biosensors must be validated to explore the effect of interfering compounds on the pesticide detection.

## Figures and Tables

**Table 1:** OP biosensors based on membrane immobilized AChE.

Mode of detection	Transducer	Enzyme immobilization method	Minimum detection limit	Linearity	Substrate/enzyme inhibitor	Time of incubation (min)	Storage stability (days)	Reference
Potentiometric	Nylon and cellulose nitrate/pH electrode	Crosslinking	0.038 *μ*M 0.077 *μ*M	50 × 10^3^–2.5 × 10^3^ 50 × 10^3^–2.5 × 10^3^ *μ*M	Trichlorfon, Co-Ral	15	30 15	[23]

Fiber-optic	Glass/sol-gel/polyvinyl	Crosslinking	0.53 *μ*M and 0.023 *μ*M	0.54–39.8 and 0.022–0.13 *μ*M	Carbaryl, dichlorvos	10	21	[24]

Amperometric	Hybrid mesoporous silica/Pt electrode	Entrapment	1.2 × 10^−3^ *μ*M	1.0 × 10^−3^–0.3 *μ*M	DZN-oxon	15	80	[25]

Amperometric	MWCNTs/PAN/Pt electrode	Affinity bonds using concanavalin A	5.0 × 10^−9^ *μ*M	3.6 × 10^−8^–3.6 × 10^−5^ *μ*M	Paraoxon	20	120	[26]

Amperometric	PAN/AuNPs/Pt electrode	Covalent Bonding	0.026 × 10^−5^ *μ*M	3.6 × 10^−7^–3.6 × 10^−4^ *μ*M	Paraoxon	20	30	[27]

Amperometric	Cellophane/AuE	Crosslinking	1.45 *μ*M	1.45–7.26 *μ*M	Paraoxon	15	NR	[28]

Dissolved Oxygen metric	Poly (2-hydroxyethyl metacrylate)/oxygen electrode	Entrapment	0.119 *μ*M	0.05–2.62 *μ*M	Aldicarb	5	2	[29]

Amperometric	PVA-SbQ/Pt electrode	Entrapment	7.2 × 10^−5^, 0.049 *μ*M	NR	Paraoxon, thifensulfuron methyl	30	30	[30]

Amperometric	Polyacrylamide/pH electrode	Crosslinking	3.62 × 10^3^ *μ*M	NR	Dichlorvos	30	50	[31]

Conductometric	Si_3_N_4_/Ti layer	Crosslinking	10 ppb	NR	Zn^+^, Cd^+^	30	20	[32]

Potentiometric	Pore glass/H^+^ electrode	Crosslinking	2 × 10^−10^ M	10^−11^–10^−4^ M	OP compounds	30		[33]

Colorimetric	H bond N^+^ membrane	Physical adsorption	1 mg/mL for omethoate, 0.1 mg/mL for dichlorvos, 2 mg/mL for methamidophos, 0.05 mg/mL for chlorpyrifos, 1.5 mg/mL for carbaryl, and 0.8 mg/mL for pirimicarb.		Op compounds	15	60	[34]

Note: NR: not reported.

**Table 2:** OP biosensors based on nonconducting polymer immobilized AChE.

Mode of detection	Transducer	Enzyme immobilization method	Minimum detection limit	Linearity	Substrate/enzyme inhibitor	Time of incubation (min)	Storage stability (days)	Reference
Amperometric	MWCNTs/PAN/Pt electrode	Affinity Bonding	5.0 × 10^−9^ *μ*M	3.6 × 10^−8^–3.6 × 10^−5^ *μ*M	Paraoxon	20	120	[26]

Amperometric	PAN/AuNPs/Pt electrode	Covalent Bonding	0.026 × 10^−5^ *μ*M	3.6 × 10^−7^–3.6 × 10^−4^ *μ*M	Paraoxon	20	30	[27]

Amperometric	MSF/PVA/GCE	Entrapment	0.2 × 10^−3^ *μ*M	0.2 × 10^−3^–44.8 × 10^−3^ *μ*M	Monocrotophos	10	30	[35]

Amperometric	PVA/SbQ/SPE	Entrapment	1.91 × 10^−2^ *μ*M 1.24 × 10^−3^ *μ*M	NR	Paraoxon and chlorpyrifos-ethyl oxon	10	Nr	[36]

Amperometric	PVA/SbQ/Pt Electrode	Entrapment	7.2 × 10^−5^, 0.18, and 0.049 *μ*M	NR	Paraoxon, maneb, and thifensulfuron methyl	30	30	[30]

Amperometric	PAMAM-Au/CNTs/GCE	Electrostatic interaction	4.0 × 10^−3^ *μ*M	4.8 × 10^−3^–9.0 × 10^−2^ *μ*M	Carbofuran	9	21	[37]

Amperometric	MSF/PVA/GCE	Entrapment	0.05 ppb (0.2 nM)	0.05–10 ppb	Monocrotophos	10	30	[38]

Amperometric	Nylon Net	Covalent bonding	1.3–3.9 ppb	NR	Paraoxon	30	15–20	[39]

Amperometric	PBA/SbQ/Pt Electrode	Crosslinking	25 ppb–1.5 ppm	NR	Chlorpyrifos	15	NR	[40]

Note: NR: not reported.

**Table 3:** OP biosensors based on conducting polymer immobilized AChE.

Mode of detection	Transducer	Enzyme immobilization method	Minimum detection limit	Linearity	Substrate/enzyme inhibitor	Time of incubation (min)	Storage stability (days)	Reference
Amperometric	Polyacrylamide/pH electrode	Crosslinking	3.62 × 10^3^ *μ*M	NR	Dichlorvos	30	50	[31]

Potentiometric	PEI/GCE	Covalent Bonding	1.0 *μ*M	NR	Dichlorvos	10	NR	[41]

Amperometric	PEI/SPE	Non Covalent Bonding	1.0 × 10^−4^ *μ*M	NR	Dichlorvos	2	NR	[42]

Amperometric	Mercaptobenzothiazole/ PANI/Au electrode	Adsorption	0.48 × 10^−3^ *μ*M 0.61 × 10^−3^ *μ*M	NR	DiazinoFenthion	20	NR	[43]

Electrochemical	PANI/CNT ssDNA/Au electrode	Covalent Bonding	1.0 × 10^−6^ *μ*M	1.0 × 10^−5^ and 1.0 *μ*M	Methyl parathion and chlorpyrifos	15	5	[44]

Electrochemical	AuNPs-PPy nanowires GCE	Entrapment	7.5 × 10^−3^ *μ*M	0.018–0.45 and 1.89–17.0 *μ*M	Methyl parathion	12	30	[45]

Amperometric	PPY-PANI/MWCNTs/GCE	Adsorption	3.02 × 10^−3^ *μ*M	0.030–1.51 and 3.027–75.67 *μ*M	Malathion	15	30	[46]

Amperometric	SF/MWNTs/GCE	Adsorption	5.0 × 10^−7^ M, 6.0 × 10^−8^ M	3.5 × 10^−6^ to 2.0 × 10^−3^ M, 1.0 × 10^−7^ to 3.0 × 10^−5^ M	Methyl parathion, carbaryl	10	4 weeks	[47]

Amperometric	CS/ALB/GCE	Encapsulation	0.86 ± 0.098 *μ*g/L	0.25–1.50 and 1.75–10.00	OP pesticides	10	15	[48]

Amperometric	PB/GCE	Crosslinking	2.5 ng L^−1^ for dichlorvos, 15 ng L^−1^ for omethoate, 5 ng L^−1^ for trichlorfon, and 10 ng L^−1^ forphoxim.	10 ng L^−1^–1 ng L^−1^ for dichlorvos	Dichlorvos, omethoate, tricholorfon, phoxim	10		[49]

Voltammetric	GnPs/Chitosan/GCE	Covalent Bonding	1.58 × 10^−10^ M	NR	Cholropyrifos	10	10	[50]

Potentiometric	Polymeric enzyme electrode	Entrapment	0–10 ppb	NR	OP pesticides	2	NR	[51]

Electrochemical	ZrO_2_-SPE	Screen printing	0.02 nM	0.05 nM to 10 nM	OP compound	40	NR	[52]

Amperometric	Au-PDDA-PB matrix	Covalent Bonding	0.8 pg/mL	1.0–1000 pg/mL and 1.0–10 ng/mL	Monocrotophos	10	30	[53]

Note: NR: not reported.

**Table 4:** OP biosensors based on sol-gel immobilized AChE.

Mode of detection	Transducer	Enzyme immobilization method	Minimum detection limit	Linearity	Substrate/enzyme inhibitor	Time of incubation (min)	Storage stability (days)	Reference
Optical	Sol-gel/TMOS	Encapsulation	0.94 *μ*M 42.19 *μ*M	3.17–31.48 14.89–998.40 *μ*M	Naled, Mecarbam	5	30	[65]

Optical	Sol-gel/Glass	Encapsulation	0.098 *μ*M	0.098–0.55 *μ*M	Paraoxon	30	NR	[66]

Amperometric	Silica sol-gel/SPE	Encapsulation	0.024, 0.015, and 0.012 *μ*M	0.01–0.001 *μ*M	Paraoxon, dichlorvos, and chlorpyrifos-ethyl oxon	20	6	[67]

Amperometric	TMOS sol-gel/SPE	Encapsulation	1.0 × 10^−3^ *μ*M	1.0 and 3.0 × 10^−3^ *μ*M	Dichlorvos	15	NR	[68]

Amperometric	TEOS sol-gel/GCE	Encapsulation	0.008 *μ*M	0.008–0.81 *μ*M	Oxydemeton methyl	20	21	[69]

Optical	Chromo-ionophore/Sol-gel	Encapsulation	2.26 *μ*M	2.26–31.67 *μ*M	Dichlorvos	15	NR	[70]

Amperometric	Al_2_O_3_ sol-gel matrix SPE	Adsorption	0.01 *μ*M	0.1–80 *μ*M	Dichlorvos	15	5	[71]

Amperometric	Sol-gel matrix on TCNQ modified SPE	Entrapment	1 × 10^−2^, 8 × 10^−4^, and 2 × 10^−2^ *μ*M	NR	Carbaryl, carbofuran, and pirimicard	20	45	[72]

Electrochemical	AuNPs-SiSG/GCE	Hydrogen bonds	0.44 *μ*M	NR	Monocrotophos	10	30	[73]

Amperometric	Alumina/sol-gel/sonogel composite/Carbon electrode	Encapsulation	2.5 × 10^−4^ *μ*M	0.5 *μ*M	Chlorpyriphos-ethyl oxon	10	50	[74]

Optical	Bromothymol blue/sol-gel	Encapsulation	0.11 *μ*M	0.14–5.70 *μ*M	Chlorpyrifos	8	10	[75]

Amperometric	Zinc oxide/sol-gel/SPE		0.127 *μ*M	0.127–5.010 *μ*M	Paraoxon	10	90	[76]

Amperometric	Silica/sol-gel/Carbon electrode	Encapsulation	3.0 × 10^−4^ and 0.47 *μ*M	3.7 × 10^−4^–1.8 × 10^−3^ and 0.27–4.09 *μ*M	Methyl parathion and acephate	20 and 4	30	[77]

Cyclic Voltametry	Sol-gel/carbon electrode	Entrapment	0.04 ppb for parathion, 47 ppb for monocrotophos	0.1–1.0 ppb	Parathion and monocrotophos	10	NR	[78]

Note: NR: not reported.

**Table 5:** OP biosensors based on screen-printed electrodes.

Mode of detection	Transducer	Enzyme immobilization method	Minimum detection limit	Linearity	Substrate/enzyme inhibitor	Time of incubation (min)	Storage stability (days)	Reference
Amperometric	TMOS sol-gel/SPE	Encapsulation	1.0 × 10^−3^ *μ*M	1.0 and 3.0 × 10^−3^ *μ*M	Dichlorvos	15	NR	[68]

Amperometric	Al_2_O_3_ sol-gel/SPE	Adsorption	0.01 *μ*M	0.1–80 *μ*M	Dichlorvos	15	5	[71]

Amperometric	Sol-gel/TCNQ/SPE	Entrapment	1 × 10^−2^, 8 × 10^−4^, and 2 × 10^−2^ *μ*M	NR	Carbaryl, carbofuran, and pirimicard	20	45	[72]

Amperometric	SPE/TCNQ/Graphite Electrode	Adsorption	3.0 × 10^−6^ *μ*M	5 × 10^−2^–0.2 *μ*M	Chlorpyrifos-ethyl oxon	10	50	[79]

Amperometric	CoPC/SPE	Crosslinking	4.9 × 10^−4^ *μ*M	10-5-1.0 *μ*M	Carbofuran	15	NR	[80]

Amperometric	Phenylenediamine/ CoPC SPE	Entrapment	1 × 10^−11^, 1 × 10^−10^, and 1 × 10^−10^ *μ*M	1.0 × 10^−11^ 1.0 × 10^−2^ *μ*M	Dichlorvos, parathion, and azinphos	10	92	[81]

Amperometric	Graphite-epoxy/SPE	Crosslinking	1.0 × 10^−4^ and 1.0 × 10^−5^ *μ*M	NR	Paraoxon and carbofuran	15	5	[82]

Amperometric	Glutaraldehyde vapour/SPE	Crosslinking	0.18 *μ*M	0.18–54.00 *μ*M	Paraoxon	10	NR	[83]

Amperometric	PVA-SbQ/SPE	Entrapment	1.91 × 10^−2^ *μ*M 1.24 × 10^−3^ *μ*M	NR	Paraoxon and chlorpyrifos-ethyl oxon	10	Nr	[36]

Amperometric	SWCNTs-CoPC/SPE	Covalent Bonding	0.01 and 6.3 × 10^−3^ *μ*M	0.018–0.181 and 6.36 × 10^−3^–0.159 *μ*M	Paraoxon and malaoxon	15	3	[84]

Amperometric	TCNQ modified-graphite	Screen printing	1 ppb	0–5 × 10^−3^ M	Methamidophos	10	NR	[85]

Amperometric	Gold electrode	Crosslinking	0.1 mM	1–10 mM	Paraoxon		28	[86]

Amperometric	Carbon electrode	Covalent Bonding	10^−10^ M	NR	Dichlorvos	60	NR	[87]

Amperometric	SPE	Copolymerisation	4 to 7 *μ*g/L	NR	Dicholrvos, methyl-parathion	4	NR	[88]

Note: NR: not reported.

**Table 6:** AChE biosensor using quantum dots as immobilization support.

Mode of detection	Transducer	Enzyme immobilization method	Minimum detection limit	Linearity	Substrate/enzyme inhibitor	Time of incubation (min)	Storage stability (days)	Reference
Electrochemical	AuNPs-SiSG/GCE	Hydrogen Bonding	0.44 *μ*M	NR	Monocrotophos	10	30	[73]

Amperometric	CdTe QDs/AuNPs/CHIT/GCE	Covalent Bonding	1.34 *μ*M	4.4 × 10^−3^–4.48 and 8.96–67.20 *μ*M	Monocrotophos	8	30	[89]

Optical	CdTe-QDs/Glass	Electrostatic interaction	1.05 × 10^−5^ and 4.47 × 10^−6^ *μ*M	1.0 × 10^−6^–1.0 and 1.0–0.1 *μ*M	Paraoxon Parathion	15	35	[90]

Fluorescence quenching	Mn: ZnSe d-dots	NR	1.31 × 10^−11^ mol	4.84 × 10^−11^ to 4.84 × 10^−6^ mol/L	Paraoxon	10	NR	[91]

Amperometric	CdTE QDs/Au electrode	Covalent Bonding	2.98 × 10^−3^ *μ*M	4.96 × 10^−3^–2.48 *μ*M	Carbyl	10	30	[92]

Note: NR: not reported.

**Table 7:** Nanoparticles based fabrication of OP biosensors with AChE as biorecognition layer.

Mode of detection	Transducer	Enzyme immobilization method	Minimum detection limit	Linearity	Substrate/enzyme inhibitor	Time of incubation (min)	Storage stability (days)	Reference
Amperometric	AuNPs/PB/GCE	Surface Adsorption	3.5 × 10^−9^ *μ*M	4.48 × 10^−3^–4.48 × 10^−2^ *μ*M	Monocrotophos	10	30	[93]

Amperometric	MWCNTs-AuNC/GCE	Hydrophilic adhesion	1.81 × 10^−3^ *μ*M	3.0 × 10^−3^–3.027 *μ*M	Malathion	8	30	[94]

Amperometric	ZrO_2_/CHIT/GCE	Surface Adsorption	1.3, 5.0 × 10^−3^, and 1.7 *μ*M	6.6–440, 0.01–0.59, and 8.6–520 *μ*M	Phoxin, malathion, and imethoate	15	30	[95]

Amperometric	Au-PtNPs/GCE	Crosslinking	50 × 10^−4^, 40 × 10^−3^, and 40 *μ*M	50–200 × 10^−3^, 1.40–50 × 10^−3^, and 40–60 *μ*M	Paraoxon ethyl, sarin, and aldicarb	25	NR	[96]

Amperometric	AuNPs/GCE	Surface Adsorption	7.0 × 10^−3^ *μ*M	28 × 10^−3^–170 × 10^−3^ *μ*M	Methamidophos	10	7	[97]

	AuNPs-MWCNTs/GCE	Surface Adsorption	1.0 × 10^−3^ *μ*M	0.1 × 10^−3^–7.0 × 10^−3^ *μ*M	NR	30	NR	[98]

Amperometric	PB/CHIT/GCE	Crosslinking	0.113 × 10^−4^, 0.703 × 10^−4^, 0.194 × 10^−4^, and 0.33 × 10^−4^ *μ*M	0.45 × 10^−4^–0.045, 0.234 × 10^−3^–0.046, 0.116 × 10^−3^–0.0194, and 0.167 × 10^−3^–0.0335 *μ*M	Paraoxon and chlorpyrifos-ethyl oxon	10	NR	[99]

Amperometric	TiO_2_-decorated graphene/GCE	Surface Adsorption	1.4 × 10^−3^ *μ*M	4.9–74.5 and 74.5–9.9 × 10^3^ *μ*M	Carbyl	3	20	[100]

Voltammetric	Graphite/CHIT/GCE	Covalent Bonding	1.58 × 10^−4^ *μ*M	1 × 10^−4^–1.0 *μ*M	Chloropyrifos	10	10	[101]

Voltammetric	MWCNTs/AuNPs-CHIT/GCE	Surface Adsorption	0.01 *μ*M	0.1–10 *μ*M	Monocrotophos	NR	50	[102]

Amperometric	CdS-decorated garphene nanocomposite	Surface Adsorption	3.4 × 10^−3^ *μ*M	9.9 × 10^−3^–9.93 *μ*M	Carbaryl	2	20	[103]

Amperometric	CHIT-GNPs/Au electrode	Chemical Adsorption	0.1 × 10^−3^ *μ*M	0.3 × 10^−3^–60.5 × 10^−3^ *μ*M	Malathion	15	NR	[92]

Amperometric	MWCNTs-CHIT/GCE	Covalent Bonding	NR	NR	Carbaryl, malathion, dimethoate, and monocrotophos	8	30	[104]

Amperometric	AuNPs/Au electrode	Surface Adsorption	33 × 10^−3^ *μ*M	10 × 10^−3^–135 × 10^−3^ *μ*M	Carbofuran	20	7	[105]

Amperometric	PbO_2_/TiO_2_/Ti	Surface Adsorption	0.1 × 10^−3^ *μ*M	0.01–20 *μ*M	Trichlorfon	10	5	[106]

Amperometric	PB-CHIT/GCE	Covalent Bonding	3.0 × 10^−3^ *μ*M	0.01–0.4 and 1.0–5.0 *μ*M	Carbaryl	10	30	[107]

Amperometric	Er-GRO/Nafion	Surface Adsorption	2.0 ng mL^−1^	5.0–100 ng mL^−1^ and 1.0–20 ng mL^−1^	Dicholrvos	10	28	[108]

Potentiometric	SWCNT modified FGE	Crosslinking	25–35 nM and 15–20 nM for darin and DFP, respectively	20–60 nM and 20–80 nM for sarin and DFP, respectively	Sarin and DFP	5	30	[109]

Amperometric	Au-PtNPs/3-APTES/GC electrode	Crosslinking	150–200 nM, 40–50 nM, and 40–60 *μ*M for paraoxon ethyl, sarin, and aldicarb	NR	Paraoxon ethyl, sarin, and aldicarb	10	NR	[110]

Amperometric	CNT-web modified glassy carbon electrode	Surface Adsorption	1 nM	20–1000 nM	Methyl parathion	20	NR	[111]

Amperometric	PAN-AuNPs	Covalent Bonding	7.39 × 10^−11^ g L^−1^	10^−10^–10^−7^ g L^−1^	Paraoxon	NR	20	[112]

Voltammetric	CdTe-GNPs film	Covalent Bonding	0.3 ngmL^−1^	1–1000 ngmL^−1^ and 2–15 ngmL^−1^	Monocrotophos	8	30	[113]

Amperometric	SiSG-AuNPs	Surface adsorption	0.6 ng/mL	0.001–1 *μ*g/mL and 2–15 *μ*g/mL	Monocrotophos	10	30	[114]

Note: NR: not reported.
